# Institutional Reform to Promote Antiracism: A Tool for Developing an Organizational Equity Action and Accountability Plan

**DOI:** 10.5888/pcd20.220368

**Published:** 2023-06-15

**Authors:** Patsy M. Polston, Derrick D. Matthews, Shelley D. Golden, Carol E. Golin, Marissa G. Hall, Emmanuel Saint-Phard, Alexandra F. Lightfoot

**Affiliations:** 1 Department of Health Behavior, Gillings School of Global Public Health, University of North Carolina at Chapel Hill; 2 Lineberger Comprehensive Cancer Center, University of North Carolina at Chapel Hill; 3 Carolina Population Center, University of North Carolina at Chapel Hill

## Abstract

Racism is a public health problem. Systems, structures, policies, and practices perpetuate a culture built on racism. Institutional reform is needed to promote antiracism. This article describes 1) a tool used to develop an equity action and accountability plan (EAAP) that promotes antiracism in the Department of Health Behavior at the University of North Carolina at Chapel Hill’s Gillings School of Global Public Health, 2) strategies that were developed, and 3) short-term outcomes and lessons learned. A study coordinator, not affiliated with the Department of Health Behavior, was hired to collect qualitative data that documented the lived experiences of students and alumni of color (ie, racial and ethnic minority students) over time in the department. Seeking action from faculty and departmental leadership, students engaged in collective organizing covered the department chair’s office door with notes describing microaggressions, and visited faculty one-on-one to demand action. In response, 6 faculty members volunteered to form the Equity Task Force (ETF) to explicitly address students’ concerns. The ETF identified priority areas for action based on 2 student-led reports, gathered resources from other institutions and the public health literature, and examined departmental policies and procedures. The ETF drafted the EAAP, solicited feedback, and revised it according to 6 priority strategies with actionable steps: 1) transform culture and climate, 2) enhance teaching, mentoring, and training, 3) revisit performance and evaluation of faculty and staff, 4) strengthen recruitment and retention of faculty of color, 5) increase transparency in student hiring practices and financial resources, and 6) improve equity-oriented research practices. This planning tool and process can be used by other institutions to achieve antiracist reform.

**Summary**

**What is already known on this topic?**

Structural racism is embedded in various systems, including academic settings. Many academic institutions are focusing time and resources on diversity, equity, and inclusion work. The challenge is that few tools exist to help address structural racism and the systems in place that contribute to inequitable policies and practices.

**What is added by this report?**

This work provides a practical step-by-step process for developing a strategic plan to guide diversity, equity, inclusion, and antiracism efforts in academic settings.

**What are the implications for public health practice?**

Our tool can be adapted and used at other institutions and organizations to address structural racism and make sustainable and equitable changes.

## Introduction

Racism has been declared a public health crisis by hundreds of US communities (1). A call for action to address structural racism has been endorsed by major institutions charged with protecting and advancing the public’s health, including the American Public Health Association (2), the Centers for Disease Control and Prevention (3), and the American Medical Association (4). Embedded in these endorsements and calls to action is the recognition that racism is a system that structures policies, institutions, interactions, and individual opportunities — and, therefore, are the drivers and determinants of health and health inequities — at all levels of the social ecological model (5–7). Although increasing attention is being paid by researchers and funders to interventions focused on structural racism as a determinant of health (8–10), the academic institutions that train public health professionals rarely have kept pace with the internal changes needed to address how the system of racism affects their own policies, structures, and practices (11,12). Reconceptualizing public health training — and the institutions that carry out this training — is essential to equip future practitioners and researchers with the skills and abilities to recognize racism and combat health inequities that stem from the effects and manifestations of structural racism (11,13–15). As a step in this direction, the Council on Education for Public Health recently revised its competencies to require all master of public health (MPH) students to “[d]iscuss the means by which structural bias, social inequities, and racism undermine health and create challenges to achieving health equity at organizational, community and systemic levels” (16). Despite this mandate from the accrediting body and increased understanding from within institutions that change needs to happen (17), there is little consensus about how to change policies and structures at schools of public health (18), a dearth of recommendations of how to train and equip faculty (19), and only a few examples of how some institutions have gone about revising their curricula and enhancing their environments with an antiracist lens (20). This article will share the process of developing a tool undertaken by a group of faculty members at the Gillings School of Global Public Health at the University of North Carolina at Chapel Hill (UNC) to generate actionable steps to promote antiracism and equitable change processes and procedures. This group offers this example and lessons learned to other institutions interested in pursuing a similar goal.

## Institutional Context

Schools of public health are embedded in and influenced by the broader history and context of their universities. UNC has a specific history that continues to permeate campus life, even with its institutional-level efforts to promote diversity, equity, and inclusion (DEI), as exemplified by the initiatives underway through Gillings’ Office of Inclusive Excellence.

Gillings has a history of activism and research that is focused on reducing inequities and advancing diversity and inclusion, and, in 2018, the school hired its first dean for inclusive excellence. The Office of Inclusive Excellence is a schoolwide office that has several full time staff who are dedicated to working to promote DEI at the school level through various trainings, workshops, and programs. In fall 2019, the school developed and adopted an Inclusive Excellence Action Plan with 6 strategic areas that focus on supporting and sustaining a diverse, equitable, and inclusive antiracist school community (21).

Alhough it is beyond the scope of this article to provide a thorough accounting of UNC’s racialized history (which would necessarily include how UNC directly benefited from the labor of enslaved people and land stolen from Indigenous tribes) or Gillings students’ efforts to address the effects of this history through its Minority Student Caucus established 40 years ago, recent events on campus provide critical insight into the context that spurred the initiatives described in this article. On August 20, 2018, the day before the academic year started, the confederate statue colloquially referred to as “Silent Sam” was toppled by activists. This event served as a flashpoint for conversations across the UNC campus that largely involved groups of students, staff, and faculty at odds with institutional leadership decisions about not just the statue, but larger issues of systemic racism. This was a culminating event that resulted from years of student activism, and although it had negative effects on student mental health (22), it also intensified the push for action and structural, not just symbolic, change.

With this resurgence of student activism around racial equity across campus, students in the Department of Health Behavior at UNC Gillings ramped up their efforts to address inequity in the department. One student group, the Equity Collective, launched a qualitative study of current and former MPH and PhD students of color in the department and shared their findings in a report in November 2019.

Department of Health Behavior faculty discussed the Equity Collective report’s findings during its February 2020 faculty meeting. The report raised concerns about the curriculum that centered on Whiteness; the focus on racism as a construct divorced from the reality of students’ lived experiences; defensiveness among faculty; and the lack of community or belonging felt by students of color in the department. From this discussion, faculty agreed a sustained effort was necessary to address the issues, rather than the ad hoc approaches attempted previously.

## Steps Toward Developing an Equity Action and Accountability Plan (EAAP)

### Step 1: Establish a team

Immediately after the meeting, 6 faculty members volunteered to work together to develop a plan to respond to the issues raised in the Equity Collective report. However, the department did not act quickly enough to communicate to students that steps had been taken to address their concerns. This lack of transparency and communication resulted in student frustration, and they organized themselves to bring immediate attention to their concerns and put forth strategies for change. On March 4, 2020, students covered the office door of the department’s chair with Post-it notes (Figure), each one communicating a microaggression experienced by a student in the department. Students also organized a collective walkout of several classes and went door-to-door soliciting commitment from each faculty member to take antiracist training.

**Figure:**
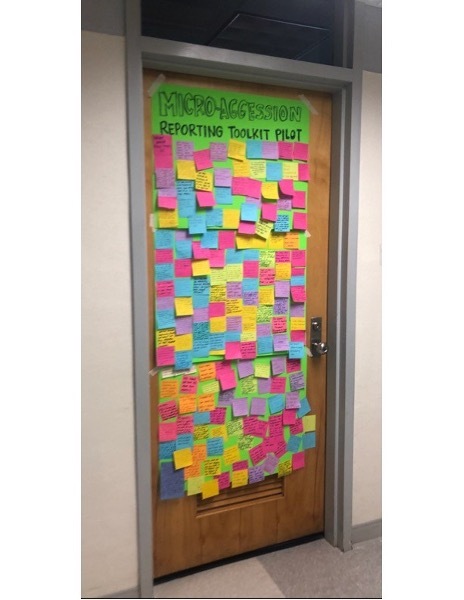
Signed Post-it notes documenting the experiences of microaggression among racial and ethnic minority students as a call to action, Department of Health Behavior, University of North Carolina Gillings School of Global Public Health, 2020. Students placed these notes on the door of the chair of the Department of Health Behavior to highlight their perceptions and lived experiences. This information became part of the input that was thematically organized by students and later incorporated in the Equity Action and Accountability Plan.

The next morning, faculty met in an emergency session to discuss ways to respond. The student action made clear the importance and urgency of the proposed work of the 6 faculty volunteers who met later that week to establish the Equity Task Force (ETF). The group (authors of this article) included 2 faculty of color (1 teaching track, 1 tenure track, both graduates of Gillings and 1 of the department), 1 tenured, and 3 assistant fixed-term professors (2 of whom have since been promoted to associate). The faculty brought varied perspectives and levels of prior involvement in antiracism and equity work, and all were fully committed to supporting students and working toward departmental change. The group convened once in person before the COVID-19 pandemic moved all school activities remotely; they met 2 hours each week during a year and a half to advance ideas for action. Described below are the process and outcomes of the work to shape a plan responsive to student demands, complementary to school-level plans developed by the Office of Inclusive Excellence, and with the potential to bring about real change in the Department of Health Behavior.

### Step 2: Identify priorities

To begin, the ETF read the Equity Collective report and the table of themes compiled from the Post-it action and elevated items that were feasible to tackle and address at the departmental level. The ETF also consulted with university and Gillings leadership, reviewed the extant literature, sought out external resources and examples of institutional change and trainings, and listened carefully to ETF faculty of colors’ own lived experiences within the department.

### Step 3: Draft Equity Action and Accountability Plan (EAAP)

The ETF outlined initial themes and corresponding action steps in spring 2020. Throughout summer and early fall 2020, the ETF solicited input to incorporate diverse perspectives into the initial draft. The initial priority areas were 1) transform departmental culture and climate, 2) enhance teaching, mentoring, and training, 3) revisit performance and evaluation of faculty and staff, 4) strengthen recruitment and retention of faculty of color, 5) increase transparency in student hiring practices and financial resources, and 6) improve equity-oriented research practices. The ETF facilitated multiple listening and feedback sessions with MPH and PhD students and provided regular updates to faculty during previously scheduled monthly meetings and a facilitated discussion session with faculty to encourage in-depth feedback and input. The ETF held a meeting with administrative and research staff to garner feedback and identify elements missing from the recommendations that spoke to staff experiences in the department. A draft EAAP was circulated by the ETF in October 2020 via the department listserv and made publicly available on the website (23) to promote transparency and accountability.

### Step 4: Incorporate input

After circulating the EAAP draft, the ETF held additional feedback sessions with 4 groups: 1) departmental faculty, 2) departmental administrative and research staff, 3) students from all the degree programs, and 4) people of color regardless of their role in the department. These meetings were focused on soliciting feedback on the content, prioritization of action steps, and plans for implementation of action steps.

The ETF also distributed a confidential online survey to all faculty, staff, and students affiliated with the department. Of 36 survey respondents, 14 were students, 12 were faculty, 7 were staff, and 3 identified as “other.” The survey assessed quantitative priority ratings of each of the draft action steps and included open-ended questions about whether any action steps were missing and whether participants had additional feedback on the draft action steps. The ETF then revised the EAAP based on feedback from the listening sessions and survey, before finalizing and distributing the EAAP in April 2021.

### Step 5: Finalized EAAP

After completing this iterative process and incorporating extensive feedback from students, faculty, and staff, a revised EAAP was produced in April 2021 (23). The 6 strategy areas in the revised EAAP were the same as those in the first draft, indicating that the original priorities aligned with the needs in the department, but the prioritized action steps evolved based on Steps 2, 3, and 4. For example, under the “enhance teaching, mentoring, and training” priority area, 4 of the original action steps remained, but 2 new steps were added based on feedback and listening sessions.

Along with a basic description and rationale for each strategy, the EAAP included short-term (<1 year) and longer-term action steps, potential barriers for implementation, and an accountability section detailing who needs to be involved or consulted (Table 1). Because the EAAP is a living document, the ETF will continue to note actions achieved. 

**Table 1:** The 6 Strategies of the Equity Action and Accountability Plan (EAAP) and Their Action Steps, Barriers, and Accountability Partners, Department of Health Behavior, University of North Carolina Gillings School of Global Public Health, 2021

Strategy	Rationale	Example action steps	Example barriers for implementation	Example accountability partners
Promote an inclusive, equitable, and antiracist culture and climate within our department.	BIPOC (black, Indigenous, and people of color) students have previously indicated not feeling supported. Enhancing the department culture and climate will help us train public health professionals who can do the same in their communities and workplaces.	Short-term: Use an equity lens to develop and disseminate a complete and accurate history of the department to students, staff, and faculty. Longer term: Develop and conduct climate surveys to assess changes over time.	Climate and power shifts in the department may be resisted by those who currently hold power.	Departmental leadership, perhaps special committee.
Boost critical reflection, training, and action among faculty to promote antiracism and equity in our teaching and mentoring.	To prepare equity-minded public health professionals, we must address equity gaps in our curriculum.	Short-term: Focus the department’s 2020–2021 annual faculty retreat on antiracism teaching strategies and skill-building. Longer-term: Review faculty syllabi and master of public health and doctoral curricula to identify equity-related gaps.	Tight budgets limit resources for teaching-related training, technologies; faculty have limited time outside current responsibilities.	Departmental leadership will need to decide to undertake trainings, but faculty will each need to adapt teaching and mentoring.
Build antiracist and equity-focused work into the performance of expectations and reviews of faculty and staff.	Antiracist actions should be both required and recognized, and support, rather than hinder, professional advancement.	Short-term: Incorporate antiracist and equity-oriented work into faculty performance planning and evaluation. Longer term: Revise promotion and tenure expectations to incorporate antiracist and equity expectations.	Performance evaluation processes are conducted once per year, with limited time to discuss many facets of faculty and staff work.	Departmental supervisors, especially department chair.
Increase diversity of health behavior faculty by improving recruitment and retention of faculty of color.	Identified goals of students, staff, and faculty; diverse learning environment better trains students for a diverse workforce; publicized racial tensions on campus may present an opportunity.	Short-term: Update faculty job posting and hiring process (including where positions are posted, application requirements, and evaluation criteria). Longer term: Commit to hiring additional faculty whose primary research area is in antiracism and equity.	Faculty hiring has a long timeline, especially when budgets are tight, making this a slow-moving goal.	Department leadership, faculty hiring committees.
Increase transparency in hiring practices for students and how financial resources are distributed.	Students have reported that hiring practices lack transparency and may be driven by connections rather than a systematic process.	Short-term: Pilot a process for requiring interviews of top candidates for Department of Health Behavior research and teaching positions before hiring decisions are made. Longer term: Create a special student jobs section in the weekly department email.	Faculty may feel responsible to fund their own mentees or people with whom they are already working rather than implement a transparent process. Not all positions may be suited to the piloted processes.	Department chair, business manager, hiring supervisors.
Enhance equity-oriented research practices, including but not limited to hiring of research faculty and staff.	Research designed to improve the public’s health must serve to dismantle inequitable structures to be successful.	Short-term: Create a repository of eligible diversity supplement grants, current research focused on inequities, examples of other health disparity research, funding opportunities for health disparity research and other resources. Longer term: Develop a set of best practices for incorporating an antiracism approach into research practice.	May require faculty time to learn and incorporate new methods; limited resources for supporting different approaches to grant writing and science.	In the short-term this can be tasked to a faculty committee, but in the long-term will require general faculty commitment, and resources for updating.

### Step 6: Implement EAAP

After developing the EAAP, the ETF generated departmental and schoolwide resources to move prioritized strategies (eg, microaggressions, faculty strengthening and accountability, mentoring) into action.

**Microaggressions.** To address students’ concerns about pervasive microaggressions, highlighted by the student Post-it action, the ETF collaborated with the Office of Student Affairs and Office of Inclusive Excellence to develop an equity-specific feedback system, Student Feedback and Equity Concerns (24). This system was incorporated into an existing general feedback section of the school’s website to elicit equity concerns, including experiences of microaggression. Language describing the new system was added to the school’s website and the template for schoolwide syllabi.

**Faculty strengthening and accountability.** One demand of the students’ collective action was that all faculty participate in a 2-day intensive Racial Equity Institute (REI) (25) training designed to help individuals (and organizations) better understand and address racism and the institutional and structural forms that have been ingrained in society. The Department of Health Behavior chair made this training a requirement, and all faculty completed REI Phase 1 training. Staff members were also supported in attending. The ETF hired an MPH student who developed a guide, Anti-racist Planning Guide for Public Health Pedagogy (26), to equip faculty with skills, resources, language, knowledge, and practices to help them examine their syllabi and strengthen their antiracist teaching practices. As another way to build the skills and practice of our faculty, the ETF organized a faculty retreat in December 2020 focused on racial equity and inclusion, which is becoming an annual event. In addition to providing faculty with new antiracism knowledge and skills and the opportunity to self-reflect, the retreat was a catalyst for strengthening the Department of Health Behavior faculty community. Finally, to enhance faculty accountability, the ETF worked with department leadership to incorporate a question into performance evaluations to assess how each faculty member contributes to antiracism and inclusive excellence in their teaching, research, service, and practice. This question is now embedded in each faculty member’s end-of-year evaluation with the department chair.

**Student mentoring.** The ETF also focused on student mentoring as a priority area. Two graduate students were hired to assist in enhancing the department’s mentoring practices, especially for students of color. One student, a male student of color and coauthor of this article, conducted interviews with students and faculty and reviewed the literature to develop a set of key recommendations to improve the mentorship experience. Building on this work, a second student sought to dig deeper into the mentorship experiences of students of color to better support them and their mentors. She conducted faculty interviews and brainstorming sessions with students of color, which resulted in a presentation and development of 2 tools: 1) a list of strategies for effective mentorship of students of color, and 2) topic items for discussion throughout the student's graduate experience, both of which are now used in Department of Health Behavior to improve the overall experiences of students in mentorship. The 2 tools described here are available on the ETF website (26).

### Step 7: Continued evolution

Developing the EAAP involved an intensive 18-month process for the 6 original ETF members and, given the importance of including new voices and garnering ownership across the faculty, the original ETF team proposed a different structure once the EAAP was launched. The ETF established Equity Action Teams (EATs) to carry out short-term steps for each strategy outlined in the EAAP. All faculty were provided with a description of each EAT and its associated strategies and were asked to indicate their top 3 choices. Using these rankings, the ETF, in collaboration with departmental leadership, assigned all faculty members to one EAT, balancing faculty diversity, team working dynamics (eg, power), and preferences. Each group also included one member of the ETF who served as a liaison between their EAT and the ETF. The ETF continued concurrently with the EATs.

Each EAT included 3 to 6 members, and its structure was decided by each group. A needs assessment was conducted by using a Qualtrics survey whereby each EAT developed questions to garner input and information needed to determine how best to carry out their action steps. Opportunities for individual EATs to report results and opportunities to seek additional faculty input took place during monthly faculty meetings throughout the academic year. Each group was tasked with completing their action steps by the end of the academic year and reporting back to the full faculty during the final faculty meeting of the school year, in May 2022. Example EAT accomplishments include the development of 1) a guide for more inclusive faculty hiring processes, 2) tips for incorporating inclusive practices in university service work, and 3) an outline of key equity-oriented research resources. Additionally, other departments at Gillings have DEI committees that are beginning to collaborate across departments and with the Office of Inclusive Excellence, which creates more continuity, efficiencies, and collectively supports efforts to advance equity, increase diversity, and cultivate an inclusive antiracist culture across the school.

### Step 8: Moving forward

Based on the key accomplishments (Table 2) of the ETF and the EATs, next steps and action steps were determined as priority items for the 2022–2023 academic year. For examples, one identified priority was to increase faculty of color in Department of Health Behavior. The department incorporated a strategy recommended by the EAT to hire an equity advocate to work alongside the tenure track search committee and to assist with the development of equity criteria.

**Table 2:** Examples of the Equity Task Force and Equity Action Team’s Key Accomplishments as of October 2022, Department of Health Behavior, University of North Carolina Gillings School of Global Public Health

Strategy	Objectives	Accomplishments	Date accomplished
Promote an inclusive, equitable, and antiracist culture and climate within our department.	Deepen faculty learning, strengthen our faculty community, and facilitate individual and collective skill-building.	A Department of Health Behavior faculty retreat in 2020 focused on antiracism teaching strategies and skill-building and was very productive and well-received, laying a foundation for ongoing exchange and skill-building.	December 2020
	Track and address microaggressions and bias-related incidents that affect Gillings students.	Supported creation and implementation of new schoolwide Student Feedback and Equity Concerns system to include fields specific to microaggressions and bias-related incidents with the Office of Student Affairs, Office of Inclusive Excellence and Human Resources.	March 2021
Boost critical reflection, training, and action among faculty to promote antiracism and equity in our teaching and mentoring.	Identify and promote opportunities for department-wide training to deepen faculty learning, strengthen our faculty community, and facilitate individual and collective skill-building.	The Department of Health Behavior implemented a new departmental policy requiring all current and incoming faculty to complete a 2-day Phase 1 Racial Equity Institute Training. Additionally, the Gillings School of Global Public Health instituted a requirement of 8 hours of equity-oriented training per year.	May 2021
	Support antiracist pedagogy and practice in public health training and education programs, including our own programs.	In summer 2020 and spring 2021, an MPH student completed a practicum with the Equity Task Force and Office of Inclusive Excellence that involved creating a guide designed to push faculty and teaching staff to examine their teaching practices and reflect on how racism, systems of power, and positionality frame our teaching. The guide was shared at the Department of Health Behavior faculty retreat and is used by the Office of Inclusive Excellence to pilot test course reviews.	Guide completed spring 2021
	Review mentoring practices, structures, and processes to better center the needs of BIPOC students and draft recommendations and guidelines that incorporate student input and best practices from the field.	In spring 2021 a first year MPH student worked with the Equity Task Force as a student-based tuition research assistant to amass resources and draft recommendations and guidelines to help the department strengthen, refine, and/or restructure its student mentoring practices. In the summer of 2021, a first-year student in the Health Equity, Social Justice, and Human Rights (EQUITY) concentration conducted her practicum with the Equity Task Force to continue this work on mentoring as well as other related tasks.	Spring and summer 2021
Build antiracist and equity-focused work into the performance of expectations and reviews of faculty and staff.	Incorporate antiracist and equity-oriented work into faculty performance planning and evaluation.	Included a question in the end-of-year faculty meeting form asking faculty to identify antiracist and equity actions taken as part of their research, teaching, and/or service during the last year. This question serves as a starting point for a discussion about ways each faculty member can continue to foster antiracism and equity-oriented research, teaching, and service.	Piloted in summer 2021, required as of summer 2022
	Encourage faculty to incorporate strategies for enhancing diversity, equity, and inclusion as part of their existing service work.	All faculty volunteered and worked on one Equity Action Team for 1 academic year to undertake tasks resulting from the recommendations in the Equity Action and Accountability Plan. Faculty on the Service Equity Action Team developed a tip sheet that provides guidance on how to enhance diversity, equity, and inclusion in current service work and made recommendations to the department to review how service work is distributed and recognized.	Completed August 2022
Increase diversity of health behavior faculty by improving recruitment and retention of faculty of color.	Update faculty job posting and hiring process. Increase transparency and communication about faculty hiring processes.	Faculty on the Faculty Hiring Equity Action Team created a hiring report with a summary of challenges in the department and recommendations for promoting equity in hiring practices.	Completed August 2022
		An equity advocate was hired as part of the search committees for hiring. All committee members are required to take specific training, including training on implicit bias. Faculty job postings in the department will now require a diversity, equity, and inclusion statement.	October 2022
Increase transparency in hiring practices for students and how financial resources are distributed.	Increase the number of open searches available to students, recommend Department of Health Behavior student positions (ie, those that are funded by the department or by grants of which an Department of Health Behavior faculty member is principal investigator) are advertised with 1) a detailed job description, 2) requirements and preferences of applicants, and 3) an application and hiring process and timeline, as possible. Distribute the postings widely via departmental listservs and weekly newsletter.	The Department of Health Behavior now consolidates student job opportunities and widely advertises through weekly departmental emails. The Equity Task Force encourages faculty to post and advertise positions at the start of each semester. The department has also initiated an annual student funding presentation and discussion for students.	Started spring 2020, ongoing
	Pilot a process for requiring interviews of top candidates for Department of Health Behavior student-based tuition–funded and teacher assistant positions before hiring decisions are made.	The department now requires interviews as part of the hiring process for research and teaching assistantships that are funded by the Department of Health Behavior, when there are multiple applicants.	Piloted in 2020, required as of 2021
Enhance equity-oriented research practices, including but not limited to hiring of research faculty and staff	Promote equity in staff hiring.	Faculty on the Staff Hiring Equity Action Team created a staff hiring report that outlines recommendations that aim to 1) promote inclusive recruitment of diverse candidates and 2) encourage inclusive and equitable candidate screening, interview, and selection processes.	Completed August 2022

Abbreviations: MPH, master of public health.

To elevate and reinforce the importance of the ETF, the committee shifted from a flat structure, in which all members had the same rank, to a hierarchal structure, in which a committee chair with salary coverage allocated by the department was selected to serve on both department and school leadership as the ETF’s inclusive excellence representative. The ETF remains active and committed to the work, which is supported by the department chair. Three students were hired to join the ETF in September 2022 to ensure that student perspectives drive the work forward. This work is ongoing and ever evolving. The members of the ETF are committed to doing this work individually and collectively to advance and promote antiracism in the Department of Health Behavior and the school.

Additionally, the ETF has been identifying ways to monitor progress, with an ultimate goal of developing systematic evaluation. At the start of the 2022–2023 academic year, the new ETF chair engaged the task force in a discussion to identify key goals for the upcoming year, with each ETF serving as a lead or co-lead for at least one goal. Each ETF meeting begins with a check-in on progress toward each goal. Each summer, other department leaders (eg, chair, vice chair, program leads) identify key objectives and measurable results and report on their progress to the full faculty at the end of the academic year; beginning in 2023, the ETF will be asked to do this as well. The long-term goal is to track the experiences of students, faculty, and staff of color, as another indicator of progress. School administrators are in the process of launching a new schoolwide Gillings Inclusive Excellence Survey; examining Department of Health Behavior–specific results will allow the department to evaluate progress while leveraging what is planned to be an institutionalized data collection tool.

## Lessons Learned and Recommendations

The context across campus, and the intensity and urgency brought by students in the department, served as a catalyzing focus for the ETF at the outset. Although the ETF’s initial charge was to respond to the student-developed Equity Collective report, the student action made it clear they would have to elevate their actions and accountability for change.

The focus of this work is for all faculty, staff, and students. The ETF responded to the concerns that primarily affected students and faculty of color that were highlighted by a diverse student body, including White students. The objective of this article is to provide an example of a tool used to encourage department-wide self-reflective work and active participation in antiracist trainings and practices that support and advance equity. For example, we described the department chair mandate to attend racial equity training. The ETF and our department chair, faculty, and staff understand that work to change systems does not fall on students alone or on the shoulders of those who are marginalized and that everyone plays a role in advancing equity.

Overall, the EAAP tool provides a roadmap and a structure for action and accountability. The ETF did not intend for this tool to be evaluated. It was created quickly, yet intentionally, out of a sense of urgency. The ETF successfully moved short-term actions items forward and developed a plan for longer-term goals. The ETF also became an example for other Gillings departments and worked with school leadership to implement some highlighted action items (eg, microaggression feedback system).

Privacy and confidentiality were not a concern in sharing lessons learned because the ETF did not provide any descriptions of participants. Instead, the ETF outlined a process for equity planning, which includes some summarizing and referencing of publicly available reports posted on the Department of Health Behavior website.

Given what was learned through the process described here, we offer key takeaway points for other institutions implementing similar efforts to bring about change. First, responding in a timely manner and being open and transparent in communications are critical. After Department of Health Behavior’s first misstep (not letting students know that the ETF was formed), the ETF provided frequent written and short updates to members of the department via a monthly newsletter and multiple in-person (on Zoom) listening and feedback sessions. Second, plans for action must be combined with measures of accountability. Third, it is essential to center the lived experiences of students and faculty of color as guideposts for change. Fourth, community members (eg, students, staff, and faculty) engaged through various methods (ie, listening sessions, surveys, and written or visual feedback) and multiple opportunities and time points throughout the process to identify needs, generate strategies, highlight gaps, prioritize action steps, and operationalize plans.

The iterative and transparent process at Gillings provided opportunities for all members of the department to be meaningfully (yet not burdensomely) involved. Students recognized the ETF’s effort and responsiveness, which helped build trust as they witnessed and contributed to the beginning of change. Faculty appreciated gaining tangible strategies and tools to enhance their pedagogy. Staff felt valued as part of the process. Although the ETF has not yet evaluated the effect of the EAAP, there is boosted commitment and movement forward in the Department of Health Behavior.

The documentation and dissemination of this process is also a sign of the ETF’s and the Department of Health Behavior’s commitment to transparency and accountability to institutional reform to promote antiracism. The work and data collected for this article were reviewed by the Office of Human Research Ethics, which determined that this submission did not constitute human subjects research as defined under federal regulations [45 CFR 46.102 (e or l) and 21 CFR 56.102(c)(e)(l)] and did not require IRB approval. Instead, we outlined a process for equity planning, which includes some summarizing and referencing of publicly available reports that are on the Department of Health Behavior’s website. In making this challenging and promising work public, the ETF hopes that the process will inspire other schools of public health to implement similar processes.
